# To Zoom or not to Zoom: A longitudinal study of UK population’s activities during the COVID-19 pandemic

**DOI:** 10.1371/journal.pone.0270207

**Published:** 2022-07-13

**Authors:** Lan Li, Ava Sullivan, Anwar Musah, Katerina Stavrianaki, Caroline E. Wood, Philip Baker, Patty Kostkova

**Affiliations:** 1 Centre for Digital Public Health in Emergencies, Institute for Risk and Disaster Reduction, University College London, London, United Kingdom; 2 EcoHealth Alliance, New York, New York, United States of America; 3 Department of Geography, University College London, London, United Kingdom; 4 Department of Statistical Science, University College London, London, United Kingdom; 5 Crisis Response, British Red Cross, London, United Kingdom; University of Almería, SPAIN

## Abstract

This longitudinal study determines the frequency and way of people doing activities from Spring 2020 to Summer 2021 during different phases of the COVID-19 pandemic in the UK. Six online surveys were carried out between April 2020 and July 2021. 4,992 participants were engaged in the cross-sectional study and 203 participants who provided repeat responses were included in the subset sample of prospective cohort analysis. Primary outcomes measured were the frequency and the mode of doing the activities (online or in-person) across sixteen selected activity groups, as defined by the UK National Time Use Survey. The results show that cultural activities, spending time with others, and travelling, were the activities with the largest proportions of frequency and mode changes. The most significant changes occurred from March to June 2020, a period that included the first lockdown. Survey results from this period show a significant decrease among most of the sixteen measured activities. From March to October 2020, a period which spans the first lockdown and its subsequent ease of restrictions, showed the most significant shift from accessing activities in-person to online. Despite ‘Freedom Day’, the July 19^th^ 2021 date in which all restrictions were abolished, it was found that people do cultural activities and group activities at a significantly lower frequency than before the pandemic. In addition, despite a lack of restrictions after this date, more than half of participants access many activities, such as spending time with others, shopping, work and studying, online or hybrid. This study provides an invaluable insight into understanding how people in the UK changed their lifestyle, including what activities they do, and how they accessed those activities in light of the COVID-19 pandemic and related public health policy implemented to address the pandemic. These results may serve as unique evidence for policymakers.

## Introduction

The virus that causes COVID-19 was first reported in human populations in Wuhan, China in December 2019 [1]. On the 30^th^ of January 2020, the World Health Organisation (WHO) characterised COVID-19 as a Public Health Emergency of International Concern, indicating the global extent of the growing coronavirus outbreak [2]. Nations were asked by WHO to take ‘urgent and aggressive action’ to curb the spread of COVID-19, and all countries were encouraged to deploy public health interventions and policies to change the course of the global pandemic at hand [3]. By April 2020, nearly 4 billion people, or half of the global population, had been asked or ordered by their governments to ‘stay home’ or otherwise limit their movements to curb the spread of COVID-19 [4].

In service to this call to action, individuals were faced with the need to modify behaviours and practises, both to adhere to new public health policies, and as a result of their own personal risk-mitigation strategies. Public health policy aimed at breaking the chain of transmission of COVID-19 by placing restrictions on elements of daily life such as travel and socialisation [5], while demanding an increased vigilance to other elements, like hygiene or health monitoring [6]. During a so-called “lockdown” behaviour change can be seen both as adherence to a type of public health policy, and as a form of resilience and adjustment to life during a global pandemic [7]. This adjusting and resilience can be seen in individuals’ use of technology to access people and activities online (through platforms like Zoom and Microsoft Teams) that were previously conducted in person [8–10].

From the emergence of COVID-19 in the winter of 2019, public health policy in the UK has intermittently mandated restrictions in movement and socialisation to curb the spread of the virus within communities [11, 12]. Considering these restrictions, one can expect behavioural change to occur in individuals across many areas of their public and private life, beyond simply what is mandated within the rules and restrictions. One widespread behavioural change many experienced throughout the pandemic has been the necessity to incorporate the use of online platforms to engage in activities remotely where one would previously have engaged in-person. An example of this is the use of Zoom to attend work meetings or to spend time with loved ones [13]. While some can seamlessly shift pastimes and livelihoods from in-person to online, other individuals may have ceased activities altogether or engaged in completely new pastimes. The dynamic nature of pandemics, including the stop-and-go cadence of the related public health restrictions, provides a complex moment in time to examine people’s behaviour [11, 12]. In understanding the activities of those in the UK during this moment, we gain insights into behaviour change, resilience, what remains unchanged during a pandemic, which would be valuable for policymakers and researchers to prepare for the future public measures and develop digital tools to support the public adapting to the pandemic [14, 15].

Within the literature, multiple research efforts took to online survey tools to measure the attitudes, behaviours, knowledge, and beliefs held during COVID-19 and lockdown [16]. Many of these behavioural studies has undoubtably found that the lockdown modifies one’s lifestyle. For instance, a few studies have found that it has affected one’s sleeping pattern [17], level of physical activity (i.e., to engage in regular or irregular exercise routines within indoor and outdoor settings) [18], and eating habits (i.e., to engage in disordered eating, or overeating) [19]. Studies on behaviour change show that across the world lockdown has modified most elements of private and public life, including change in sleep, livelihoods, learning, mental health, movement, and hygiene [17–19].

On January 30^th^, 2020, the first cases of COVID-19 were confirmed in the United Kingdom [20]. Prior to these confirmed cases, the Department of Health and Social Care and Public Health England had been reporting rising risk levels from ‘very low’ to ‘low’, reflecting the emerging threat of the spread of the virus. Throughout March, especially in light of the first death to occur from the virus within the UK, the threat levels rose from moderate to high, and policies began to be implemented to actively contain the spread of new cases [21]. On March 23^rd^, 2020, the Prime Minister of the United Kingdom announced the first ‘lockdown ‘in the United Kingdom, ordering people to ‘Stay Home, Protect the NHS, Save Lives’ [22]. On March 26^th^, 2020, the ‘Health Protections (Coronavirus, Restrictions) (England), 2020’ officially commenced the first COVID-19 related lockdown in England, with expectations that the lockdown would continue for “at least three weeks” [23]. With this announcement, enforceable restrictions on movement, gatherings, and business closures were set forth. This ‘first lockdown’ ensured that all public gatherings of more than two people were prohibited and leaving home required a ‘reasonable excuse’ including the procurement of essential services [23].

The enforced lockdown by the UK government will undoubtably have some positive or detrimental effect on the daily lifestyle routine on the population as a whole as evidenced in previous studies [17–19]. We therefore deployed a series of online surveys to understand the impacts and how people in the UK spent their time from January 2020 to October 2021 broken into 7 phases (as defined below), as well as the chosen mode of access to these activities, whether online or in-person. The possibility to perform activities online may allow the frequency of many activities to remain high during the COVID-19 restrictions. It could be expected that many activities undergo a shift in the mode of access from in-person to online during times of restrictions. What is less understood is a more granular look at an individual’s behaviour as restrictions and mandates constantly change throughout the time periods.

**Fig 1:**
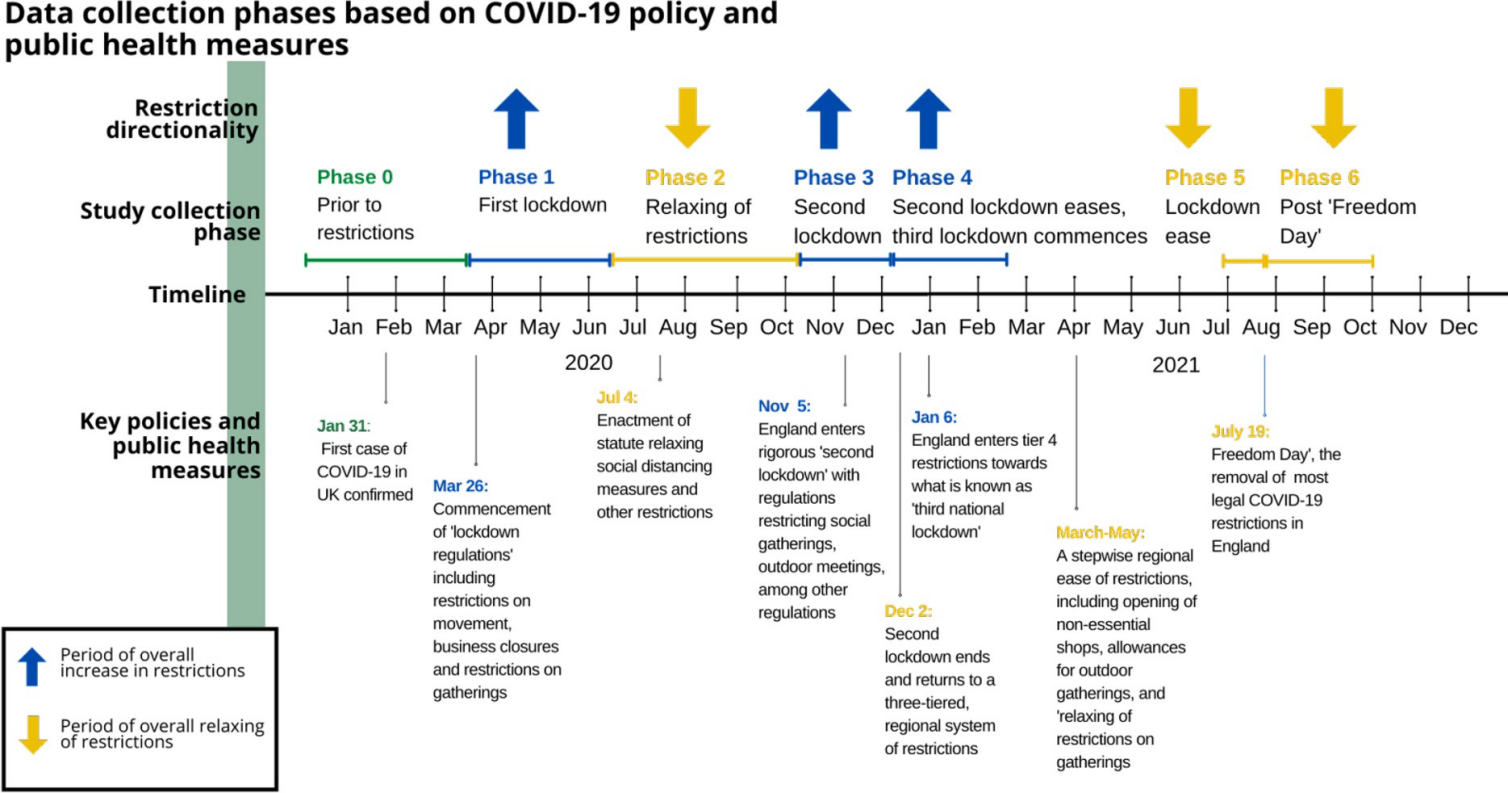
Data collection phases based on COVID-19 policy and public health measures.

To bridge this gap, this study will focus on the frequency and the way of people doing daily activities throughout the pandemic. Fig 1 shows how ‘study phases’, that is, when versions of the survey were administered, relate to changes in COVID-19 policy in the UK. The first lockdown in England lasted well beyond the original estimation of three weeks; the announcement of relaxing restrictions occurred on June 23^rd^, 2020 [24] and included new mandates such as enforceable mask-mandates, as well as limits on movement and gatherings, especially related to indoor settings [25, 26]. Over the following months, the UK was faced with two further lockdowns, with relaxing restrictions occurring piece-meal between each period of lockdown [22]. Furthermore, guidelines and policies were being developed and implemented to address regional or local hot spots of COVID-19 beyond nationwide lockdowns. For example, in a period of relaxed nationwide restrictions, local restrictions implemented based on caseloads of regions with Scotland, England, and Wales, has nearly 3 out of every 4 UK residents under increased restrictions, despite not being under a widespread ‘lockdown’. Whether during a nation-wide lockdown, or regional or city-wide mandates, individuals were asked on a weekly basis to adjust their movements, behaviours, and livelihoods to curb the spread of COVID-19.

## Methodology

A longitudinal survey was conducted in which participants were recruited through online channels by means of online advertising. The snowball approach was our sampling strategy to recruit as many respondents as possible.

### Questionnaire design

The questionnaire seeks to measure the change in individuals’ behaviours prior to, and during, the six phases of the COVID-19 pandemic as defined above. The original questions were composed of three sections with 48 items in total.

In the first section of the survey, the questionnaire seeks to measure the frequency and mode (online or in-person) of activities performed at the time of the survey. The survey measures 16 activity types using categories defined by the UK National Time Use Survey [27]. The activities included in the survey are listed in Table 1. Participants were asked two questions for each of the 16 activities; the frequency they performed the activity and mode of access. When new participants enrolled in the study, questions were also included that aimed at measuring activity frequency of the past, before the COVID-19 restrictions in the UK (phase 0).

**Table 1:** Activity type and examples.

Activity name	Example (provided in questionnaires)
Group activities	Doing group activities with others e.g., sports teams, exercise class, religious meetings, art class
Cultural activities	Cultural activities, e.g., going to the cinema, concerts, visiting libraries, museums
Spending time with family	Spending time with whom you live with e.g., family, partner, roommates
Spending time with others	Spending time with others who you do not live with e.g., extended family, friends, colleagues …
Relaxation	Relaxing and taking time out, e.g., meditation, resting
Getting active	Getting active, e.g., walking, running, going to the gym, cycling, swimming
Interests	Taking time for your personal interests/hobbies, e.g., surfing the internet, gaming, playing a musical instrument, creative writing
Journaling	Journaling, e.g., handwritten, typed, art journaling, online blogging, etc
Social media	Using social media networks, e.g., Facebook, Instagram, Twitter, LinkedIn etc.
Word affairs	Keeping up to date with the world affairs, e.g., watching TV, listening to the radio, reading the news, etc
Housework	Doing work on and around your home, e.g., cleaning the house, home improvements, DIY
Shopping	Shopping habits e.g., groceries, household items, clothing, non-essentials
Work and study	Working or studying, e.g., employment, studying at college or university for a qualification
Traveling	Commuting to and from work/study, e.g., going to a specific place to undertake a job
Helping others	Helping others, e.g., volunteering, freely offering to help a person, group, or organization
Pets	Looking after a pet, e.g., walking your dog or someone else’s

The second section of the questionnaire included questions to measure participants’ demographic background (e.g., age, gender, education, employment, household number and characteristics of the participant’s household). This section also included four questions regarding the participant’s COVID-19 infection and testing history. In the third section, questions measured emotional status through a Positive and Negative Affect Schedule (PANAS) [28]. While all three sections remained present across each questionnaire administration phase, each survey varied slightly to address changes across the public health situation. For example, three vaccine-related questions were added to the questionnaires starting with phase 4. A comprehensive version of the questionnaire that includes all questions across each survey version is included in S1 File.

### Recruitment and data collection

The survey was administered online over a fifteen-month period (April 2020-July 2021). The survey was administered six distinct times, called study ‘phases’, over the course of the pandemic. The first survey was advertised to the general public through Facebook, mutual aid groups, and other social media channels, with the aim to recruit randomly from those 18 years or older. The first survey was taken by 3,240 participants, who also voluntarily provided their email addresses. Using the email list provided by the first round of surveys, the five follow-up surveys were carried out in May 2020 (n = 1399), October 2020 (n = 856), December 2020 (n = 1050), June 2021 (n = 1298) and July (n = 1036). New participants were added for the third survey in October 2020 (n = 1762) and the fourth survey in December 2020 (n = 143) through Facebook advertising. All the survey data was collected via SurveyMonkey.com. As we were seeking comparative analyses to ‘normal = pre-pandemics behaviour, we introduced so-called phase 0 (prior to the pandemic) asking participants how their behaviour changed in comparison to this baseline.

### Data interpretation and cleaning

The surveys collected were divided into seven data-collection phases characterised by the public health restrictions occurring in the UK over the course of the study period, as shown in Fig 1 and Table 2. The data in phase 0 was extracted from surveys running in April, October, and May when the new participants were added and the questions about the lifestyle prior to lockdown were asked. The records that spanned the node of phases were re-categorized based on the collection date. In each phase, the participants answer questions regarding the sixteen activities. For each activity, two questions were asked; that of measuring frequency (“At the moment, how often do you do…”) and mode (“how do you do these types of activities compared to before COVID-19?”). The frequency was measured by 6 ordinal options from “never” to “more than once per day”. The mode was measured by four options including “nothing changed” (comparing to the way before the pandemic, which is assumed as fully in-person), “some online and some in-person”, “fully online” and “stopped”.

**Table 2:** Number of records in each phase and related policy.

Phase Number	Collection period	Number of respondents	Policy marker
Phase 0	Prior to March 23^rd^, 2020	4604	Prior to the pandemics and COVID-19 related restrictions
Phase 1	March 24^th^, 2020, to June 20^th^, 2020	3505	Period of overall increase of restrictions, including commencement of first national lockdown
Phase 2	June 21^st^, 2020 to October 14^th^, 2020	381	Period of overall relaxing of restrictions, including the reversal of first national lockdown restrictions
Phase 3	October 15^th^, 2020, to December 2^nd^, 2020	2212	Period of overall increase of restrictions, including commencement of second national lockdown
Phase 4	December 3^rd^, 2020, to February 22^nd^, 2021	1146	Period of overall increase of restrictions, including commencement of third national lockdown
Phase 5	April 4^th^, 2021, to July 18^th^, 2021	1227	Period of overall relaxing of restrictions
Phase 6	July 19^th^, 2021, to August 15^th^, 2021	969	Period of overall relaxing of restrictions, including reversal of most nationally mandated COVID-19 restrictions

Two different study designs have been used to interpret and analyse the survey data. First, to understand how people changed their behaviour in the six phases, a cross-sectional design study was used to analyse the whole sample. Second, a prospective cohort study design was used to explore how individuals were adapting to the pandemic throughout the phases using a subset of paired samples. Only those who had participated in all, but the second survey were included in the second analysis.

To avoid double-counting responses, all duplicate email addresses were assessed in each phase. Upon deduplication and the removal of incomplete records, there were 4,992 unique participants included in the cross-sectional study and 203 participants who participated continually (phase 2 were excluded due to insufficient sample size) were included in the prospective cohort study.

### Data analysis

A series of statistical tests were used to understand the patterns of behaviour related to the sixteen activity types during the six different phases of lockdown in the United Kingdom. Different sets of statistical tests were deployed for the cross-sectional study and for the prospective cohort study, to understand both the change of individuals over time, and a general look at the group at-large during a single moment in time.

For the cross-sectional study, descriptive analysis was performed by drawing stacked bar plots for each activity to visually assess the data distribution. Next, a series of Chi-squared tests and Fisher’s exact tests were carried out in a phase-by-phase manner (e.g., Phase 2 versus Phase 1, and Phase 3 versus Phase 2, etc.,) to determine whether there was a significant difference in the frequency and mode separately. Our null hypothesis is that the proportions of the frequency or mode in each activity between two phases are the same, thus each test result indicates if there is a significant change or not. Word clouds were generated to get a generalised sense of the write-in data in order to inform context for analysis.

For the prospective cohort study, the frequency data from “never” to “More than once per day” was firstly coded from 0 to 5 so that a one-sample Wilcoxon signed-rank test could be used to determine whether there were any significant differences in the frequency of the activities and whether the levels of such frequency were increasing or decreasing, from phase to phase. We first hypothesised that the medians of frequency change in the adjacent phases are equal (Null hypothesis), and consequently the result indicated whether there is a significant change between the two phases. To interpret the results for the Wilcoxon paired test, the tests were supplemented with graphical outputs using Alluvial plots to show the respondent’s change in frequency across phases. These patterns are illustrated by the flows in the Alluvial graphs. The McNemar-Bowker test was used to understand whether there is a significant change in the mode of doing the activities on a phase-by-phase basis with the hypothesising that the proportion of the mode change of two adjacent phases is equal (Null Hypothesis). All tests are deemed statistically significant if the p-value is less than 0.05. All statistical tests were carried out on the RStudio desktop version 2021.09.1+372.

### Ethics statement

The authors assert that all procedures contributing to this work comply with the ethical standards of the relevant national and institutional committees on human experimentation and with the Helsinki Declaration of 1975, as revised in 2008. University College London Ethics Committee (code: 4147/002) approved all procedures involving human participants. Informed consent for participation in this project was provided by respondents prior to the beginning of the survey. Participants provided informed consent which was recorded digitally on the survey platform (Microsoft Forms). This study did not include minors.

## Result

In this section, we started with providing the sociodemographic characteristics of the sample included for the cross-sectional study and the subset sample of the prospective cohort separately, then presented the exploratory and analytic results of each study with graphs and interpretations.

### Sociodemographic characteristics of study participants

The total cross sectional study sample was comprised of 4,992 participants. Of those who provided demographic information (n = 4,222), 83.4% participants were female (n = 3,521), and 14.4% were male (n = 650); 62% were aged over 55 (Range 55–64: n = 1414, 33.5%; Range 65+: n = 1204, 28.5%); and most of the participants were white (n = 4030; 95.4%). Most of the participants had a high-level education: 46.8% with bachelor’s degree (n = 1977), and 31.6% with post-graduate degree (n = 1334). Around 55% were employed or self-employed (n = 2328), and 32.8% were retired (n = 1383). Most of the participants were living in the south part of England at the time of the survey (Top 3 regions: Southeast 22.52%, London 13.50%, and Southwest 10.59%) among all the participants. These participants completed the surveys once or multiple times, which contributes to different numbers of records and demographic compositions in each phase. The socio-demographic details of each sample can be found in S1 Table.

203 people participated in the surveys continuously (apart from phase 2) and were able to be analysed as a prospective cohort study. 199 of this cohort reported their demographic information. Around 70% are aged over 55, 39.60% of whom are aged over 65. The majority of the participants are female (81%), and white (96.57%) with higher education backgrounds (bachelor’s degree: 57.72%; post-graduate degree: 34.48%). About half of them were employed or self-employed (n = 101, 49.75%), and 41.87% were retired (n = 85).

### Activity changes throughout the seven phases of the pandemic

**Fig 2:**
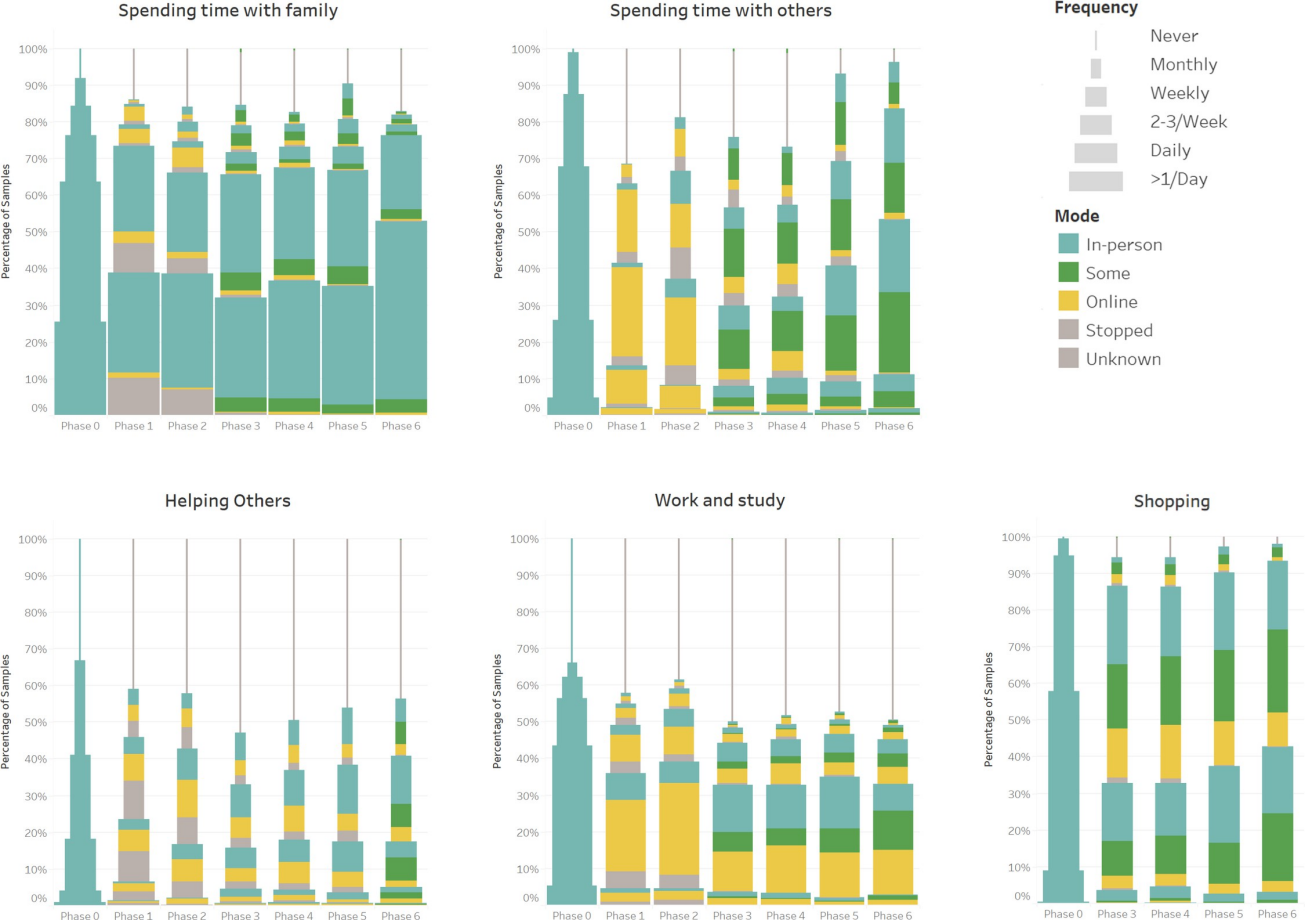
Stacked bar showing the frequency and mode changes of ten activities (1–5) across 7 phases; the modes in phase 0 were assumed to be all doing in-person.

**Fig 3:**
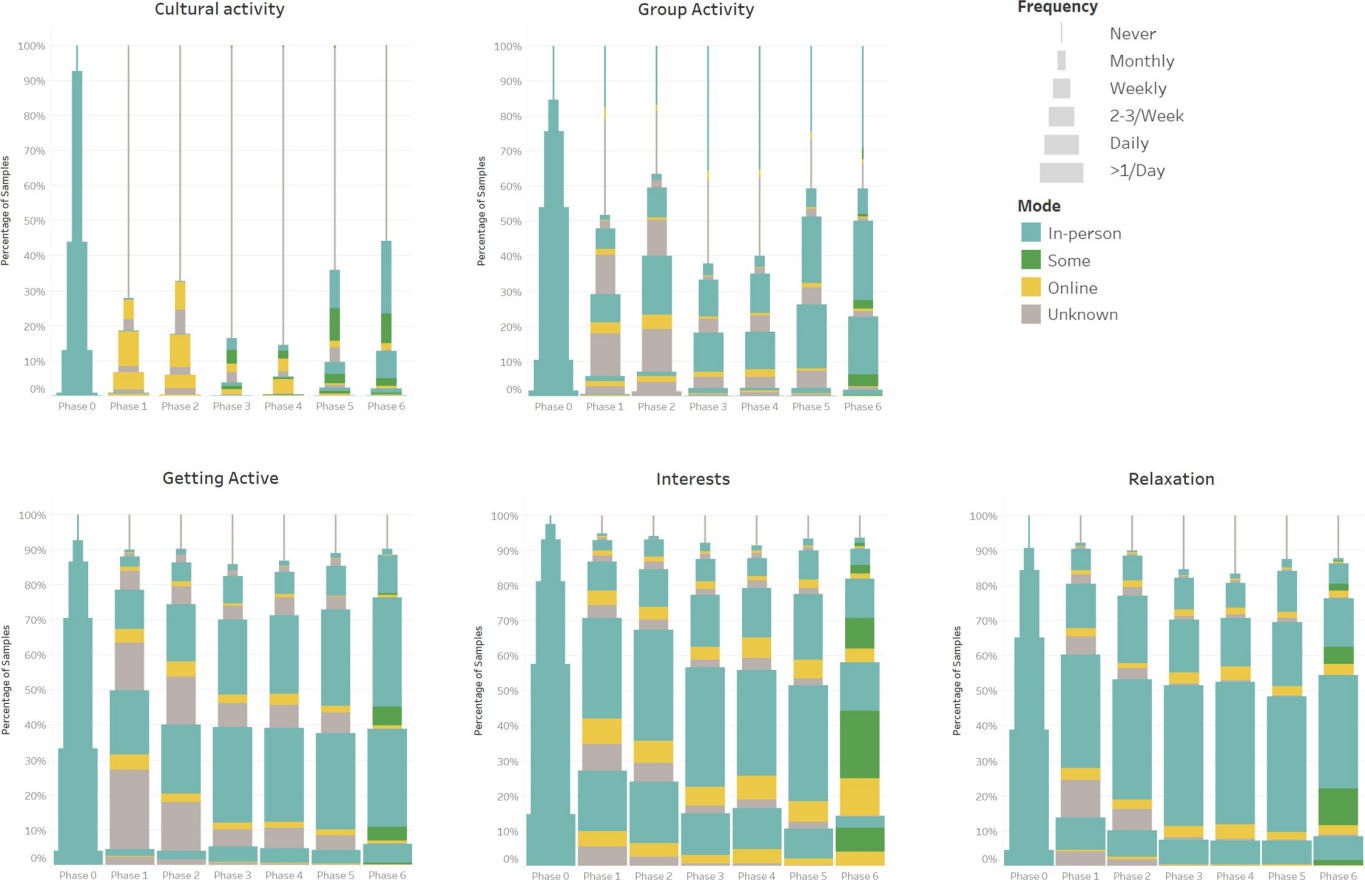
Stacked bar showing the frequency and mode changes of ten activities (6–10) across 7 phases; the modes in phase 0 were assumed to be all doing in-person.

**Fig 4:**
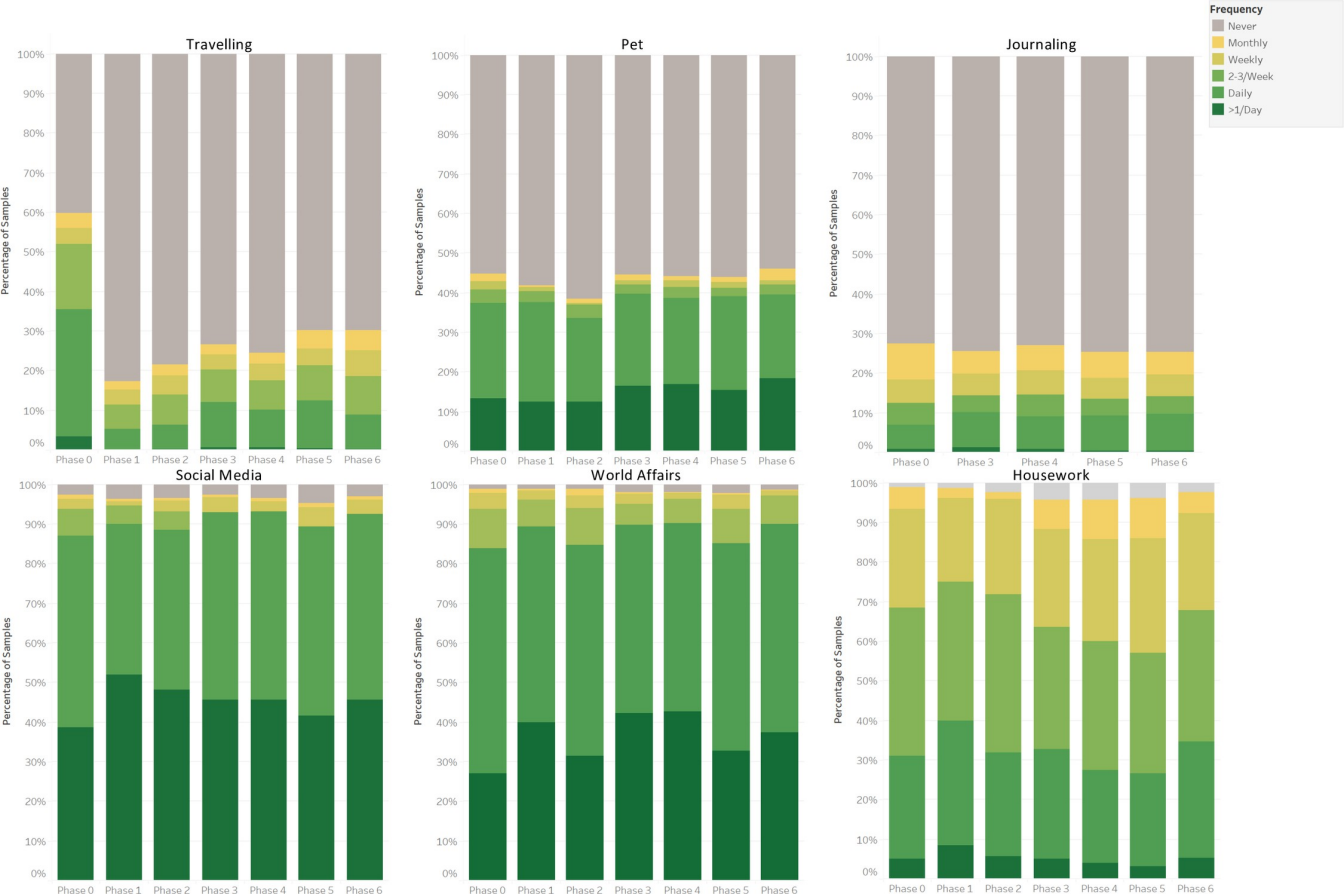
Stacked bar showing the frequency changes of six activities across 7 phases; P-value has been calculated by Chi-square goodness of fit test.

To measure the change of behaviours due to the pandemic, the outcomes measured were the frequency (6 Likert-scale options from “never” to “more than once per day”) and mode (in-person, online, some online or stopped) each activity. Based on the nature of each activity, frequency and mode were measured for ten activities, and six activities we measured frequency only (Figs 2–4). To show the changes statistically, we calculated the frequency and the proportion change by phase to phase (See S2A Table). The Chi-square goodness of fit test and Fisher’s exact test were used to test the significance for frequency and mode changes in each activity, respectively. All the statistical test details were fully reported in S2B and S2C Table. In this section, we summarised the result in each phase.

From phase 0 to phase 1, cultural activity (Monthly, -39%; Never, +67.9%), travelling (Daily, -26.5%; Never: +46.3%), and spending time with others (2–3 times per week: -12.4%; Never: +31.6%) were largely affected since the pandemic started and the frequency declined sharply (all p<0.001). Most of these activities turned online from phase 0 to phase 1. The frequency of doing group activity also dropped markedly (2–3 times per week: -18.9%; Never: +34.8%, p<0.001), but only a small proportion (6.5%) shifted to online. We can see there are slight increases among doing housework and keeping up with the world affairs. The frequency of other activities all presented a slight decline trend and mostly in person (all p-value < 0.001).

There is a slight increase in frequency from phase 1 to phase 2 among cultural activities (Monthly: +5.3%; p<0.001), group activity (2–3 times per week: +8.6%; p<0.001), spending time with others (Monthly: +8.7%; p<0.001), work and study (p>0.05), and travelling (p>0.05). However, the declined trend kept in relaxation, spending time with family, helping others, keeping pets, getting active, and doing housework. The way people do the cultural activity is still nearly all online, but more were shifted to online among spending time with others, work and studying, group activity. The other activities presented similar trends to those from phase 0 to phase 1.

In phase 3, more people do these in a mixed way—some in person and some online. Meanwhile, the frequency of doing most of the activities showed a significant decrease, especially in the group activity (2–3 times per week: -17.1%; Never: +25.7%; p<0.001), cultural activity (Never: +16.5%; p<0.001), spending time with others (Never: +5.5%; p<0.01), and work and study (Never: +11.6%; p<0.01). However, the exceptional activities are travelling, keeping pets, and using social media, which showed a slightly increasing trend.

In phase 4, most of the activities presented a similar result in phase 3 (only spending time with family and others, doing housework displayed significant change), but they were changed strikingly from phase 4 to phase 5. Nearly all the activities were done more frequently and more in person, particularly among spending time with others, cultural activity, and group activity, but there are slight decreases among journaling and using social media.

The difference between phase 5 and phase 6 is small, although a slight increase in terms of the frequency and using the in-person mode was shown among all the activities except for work and study, which was steady since phase 3 and displayed a slight decrease from phase 5 to phase 6 (p>0.05).

Additionally, the “what new activities you have done recently” question was added to the questionnaire under ten activities in phase 6. Five answers are valid for analysis and interpretation, while the other five are full of noise. Based on the qualitative result, we can know that compare to the behaviour before the pandemic and during the lockdown, in phase 6, the new cultural activities that people started to do are visiting the theatre, going to the cinema, and attending concerts; the new group activities are group meetings, exercise classes, choir, swimming, and dancing; the new activities for getting active are swimming, going to the gym and walking; new hobbies are choir, painting, yoga and walking; new activity for relaxation are yoga, walking and mediation.

### Individual adaptations of activities throughout the pandemic

Respondents who participated in the survey continuously over time allowed researchers to gain a comprehensive picture of their changes in behaviour during the various stages of the pandemic.

**Fig 5:**
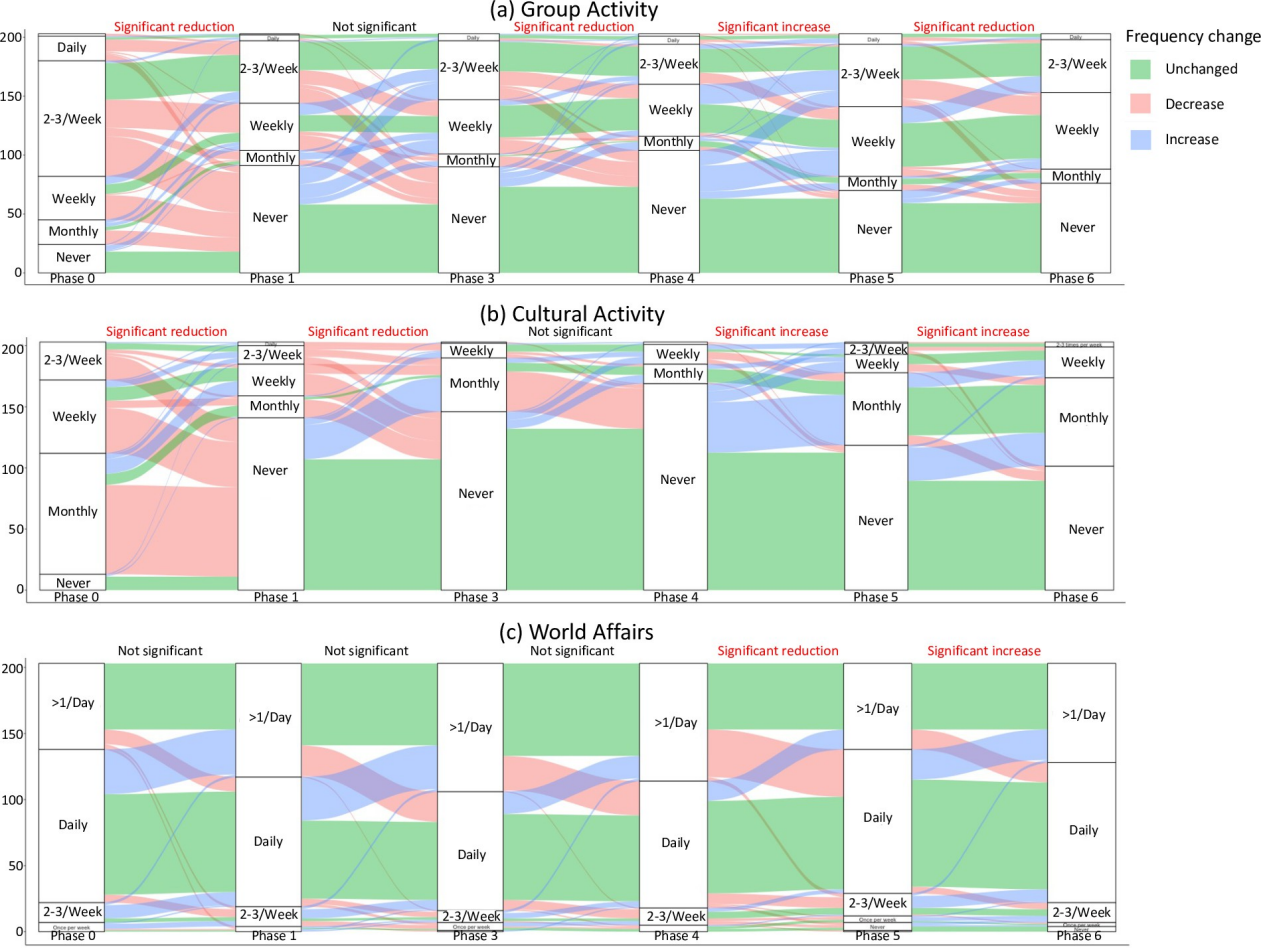
Examples of alluvial plots indicating frequency change of people performing group activity, cultural activity and keeping up with world affairs among the repeated measures (N = 203) throughout the five phases (phase 2 was left out of the analysis due to insufficient samples); P-values were calculated by one-sample Wilcoxon signed-rank test to determine significance in changes on phase to phase basis; see S2 File for all activities.

The Alluvial diagrams (Fig 5) illustrate the changes in how respondents engaged with a particular type of activity in terms of frequency, and whether such patterns significantly increased (blue), decreased (red) or remained neutral (green) across the six phases of lockdown. The alluvial plots allow us to see change in frequency dynamically across the study period. For instance, when considering the example of Group Activities (Fig 5A), we found that many of the respondents were engaged in this type of activity for at least ‘2–3 times per week’ prior to lockdown (phase 0). However, we found a significant reduction in the frequency for Group Activities as soon as lockdown kicks-in in phases 1 and 4 (*p* < 0.05) where a substantial number of respondents would cease such group-related activities in lockdown by reporting ‘Never’. In phase 5, it depicts a significant increase in Group Activities which show most of the respondents transitioning from ‘Never’ to ‘Once per week’ or ‘2–3 times per week’ (*p* < 0.05), an indication that respondents are resuming their once regular routines in phase 0 as the country re-opens from lockdown. Let us consider another clear example for Cultural Activities (Fig 5B), we observed that many respondents were engaged in this type of activity on a ‘monthly’ basis (followed by ‘once per week’ and ‘2–3 times per week’). However, as soon as the lockdown starts, we see a substantial plummet in Cultural-related activities through phase 1 and 3 (*p* < 0.05). When the lockdown in phases 5 and 6 are easing, we can see that the frequency in cultural-related activities starts to confidently pick up, whereby such increase is statistically significant (*p* < 0.05). One activity that remained neutral across all phases for obvious reasons regardless of significance were activities relating to respondents keeping up with World Affairs (Fig 5C). Almost the entire cohort of respondents was engaged with this type of activity on a ‘Daily’ or ‘More than once per day’ basis, and the lockdown phases is associated with little to no interruption to this type of activity–for obvious reasons, where the global pandemic crises and lockdown situation was dominated in most news outlets in the UK (i.e., TV, radio, newspapers etc.,); these respondents were keeping up to date with such information. All the statistical test details were fully reported in S3A Table and the other plots for remaining can be viewed in S2 File.

**Fig 6:**
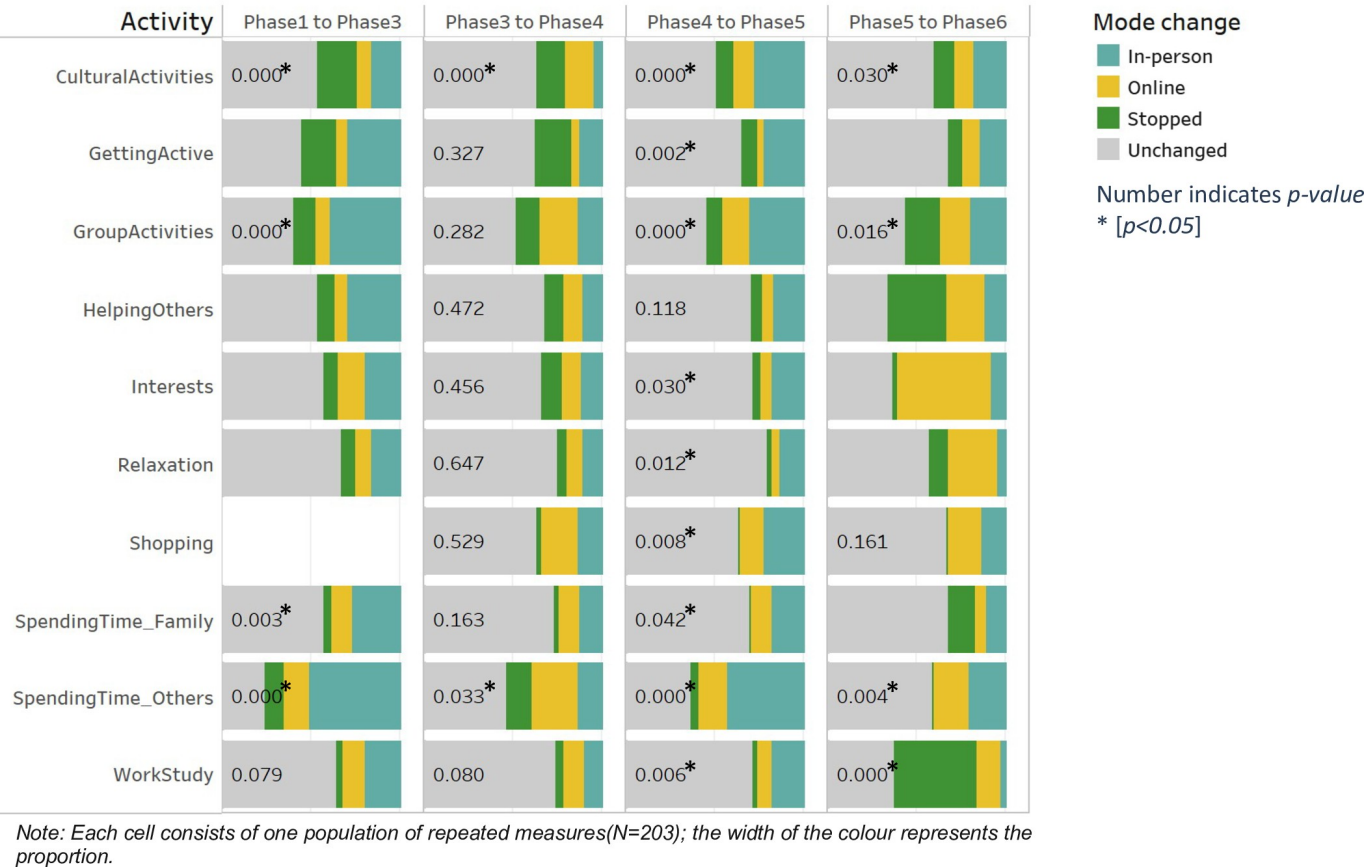
McNemar-Bowker test for mode change among the repeated measures; each cell represents the total repeated measures (N = 203); the width of the colour represents the proportion; phase 2 was excluded due to insufficient sample.

In terms of the mode change (either shifting to more online or shifting to more in-person) in the repeatedly measured sample, the McNemar-Bowker test has been used and the result is shown in Fig 6 (the test result details were fully reported in S3B Table). From Phase 1 to Phase 3, there is a significant change in the way people do culture activity (p<0.001), group activity (p<0.001), spending time with family (p = 0.003), and spending time with others (p<0.001). Of these, the ways of people spending time with others and family and doing group activity were mostly changed to in-person, while the ways of people doing cultural activity were mostly stopped. From Phase 3 to Phase 4, there is a significant change in the way people do cultural activities (p<0.001) and spend time with others (p = 0.033). The way people spend time with others was mostly changed to online, while the way of people doing cultural activity mostly moved to stopped and online. From Phase 4 to Phase 5, there is a significant change among all the activities except for helping others. All these changes were mostly moved to in-person. From Phase 5 to Phase 6, there is a significant change in the way people do culture activity (p = 0.03), group activity (p = 0.016), spending time with family (p = 0.004), and work or study (p<0.001).

## Discussion

To our knowledge, this is one of the first study to implement a hybrid of study designs to explore the impact of the lockdown on the daily lifestyle routines on the British population. It is unsurprising that the results of the survey measure difference in types of activities and modes of access to activities across various phases of the pandemic. The use of two study designs and the corresponding analytic approaches solicits results that address different aspects of the research question at hand and allow the authors to answer questions about the frequency of different activities, and where people were accessing these activities online or in person.

### Activity changes throughout the pandemic

The cross-sectional study results show that cultural activities, spending time with others, and travelling, were the most affected activities across the entire study period. These activities are related to social togetherness and movement, both of which were explicitly restricted at times by public policy, and were also elements of behaviour that, despite specific restrictions, people were being implored to limit. A decrease in cultural activities, such as going to a museum or the movies, as well as travelling and spending time with others, is an activity that dropped most significantly during the entire study. As can be seen, the few respondents that kept up these activities often shifted to accessing these activities online. The finding that these are the activities most affected during the pandemic is worth understanding for many reasons. Firstly, it is important to understand the mental health repercussions of such a seismic shift in social connectedness. In addition, the industries associated with these activities, like travel and live entertainment, will need to look toward crisis and contingency planning to prevent devastating losses during future crisis.

In terms of both frequency and mode, the most significant change occurred between phase 0 and phase 1 of the pandemic, with a significant decrease occurring across many activities. This period corresponds with the earliest period of the pandemic for those in the UK. While this is a period that corresponds with increasing restrictions, including the first national lockdown, it does not necessarily capture the most rigorous policy-based restrictions, despite this large, measured decrease in activities. This large activity decrease may be associated with our early understanding of the pandemic and may also capture the extreme nature of our uncertainty and fear during this time. Additionally, it represents a period where compliance may be at its highest before people become burned-out or hesitant to restrict their actions. The finding that this period represented the most significant change, despite it not holding the highest level of restrictions, shows an area for risk communication improvement. Fear and panic should always be mitigated during communication to the public. Those communicating risk, such as policy makers and government officials, should be able to address the fear and uncertainty within the community at the beginning of a crisis. In addition, the beginning stage of a long-term crisis could be considered a potential point of high compliance and impact for behaviour change.

Most of the sixteen measured activities during phase 0 to phase 2 shifted from being conducted in-person, to being accessed online. This shift illustrates the increasing use of online platforms by citizens, which many of us became accustomed to by October 2020, the end of phase 2. By this time, phrases like ‘Zoom fatigue’ had already begun to creep into our ‘new normal’ vernacular [13]. It is worth noting that although during a short period of easing restrictions in the summer of 2020 (covered in Phase 2), this trend was still shaping and growing. It appears that many activities similarly swung back to being conducted in-person or in a hybrid way (both in-person and online) during the shift from phase 3 to phase 5.

Looking overall at the cross-sectional results, most people made a drastic shift across all activities towards online access during Phase 0–1, whereas regardless of policy, the subsequent phases remained more mixed. The mixed nature of the access of these activities in the later part of the pandemic may show people’s confidence with making independent personal decisions about their time-spent online and in-person, as pandemic life became more familiar. Despite the mixed mode of access, in general, there was a decrease of activities across most activities during phase 3 of the study.

Generally, we can see that the pandemic, and its policies, changed how people spent their time in the UK. Between phase 0 and phase 6 when all the restrictions were abolished, people still maintain the habits shaped during pandemic and lockdown, which is in accordance with other research findings where behaviour change was sustained [29]. For example, respondents tended to do cultural activities, get active, and perform group activities less frequently, and accessed those activities online. Similarly, it was found that more than half of participants who accessed their interests, spent time with others, shopped, worked, and studied online or in a hybrid way in phase 6. The choices are probably affected by age, gender, education, and employment conditions and may also have an impact on their mental health status. We will explore these hypotheses in follow-up studies.

### Individual adaptations of activities throughout the pandemic

Analysing the data using prospective cohort approach allows researchers to understand the change in activities of a specific group over the study period and allows a look at the specific temporal sequence of events. In the case of this study, it allows us further clarity as to what the effect of timing of the pandemic environment did on people’s activities and the use of in-person or online outlets for their day-to-day engagements. Within the cohort, we can see a large decrease in frequency across many activities during Phase 0 to 1. Activities such as group activities, travelling, cultural activities and spending time with others dropped significantly in all groups, regardless of whether people had previously frequently or infrequently done these activities prior to the pandemic. This is unsurprising, as this phase represents the most jarring of time periods, a period where uncertainty and fear were rampant. This also represents a time in which people may have been unsure of how to access activities online, a way of continuing with favourite activities during stringent lockdowns. Some activities, regardless of timing within the pandemic, did not experience this drop in frequency across the cohort. Pastimes like social media viewing, watching world affairs, tending to pets, and other home activities were unsurprisingly consistent across periods of the pandemic when restrictions were high.

Some activities are shown to drop in the early phases of the pandemic but begin to increase in frequency before restrictions on movement and socialization were fully lifted. This can be seen in pastimes like cultural activities and spending time with others, where there is a significant drop in frequency between phases 0–1, and 0–1, but the frequency begins to rise again in 3–4. One explanation for this might be policies that were developed before all restrictions were listed (within phase 6) which allow people to access activities safely and comfortably while still following restrictive public health policies. Examples of this include limited, timed-tickets museum visits, or spending masked, socially distanced outdoor time with others. These are strategies and methods that arise out of human resilience during pandemics that allow for simultaneous experiences of achieving desired pastimes with compliance with public policy measures.

What we can also see from the prospective cohort study is the changes related to temporality in online and in-person activities. The results of the study show that there are some activities that individuals are generally okay with performing online, or at least occasionally so. The results of the study show that for some activities, despite the level of restrictions in place, people remained to access online, such as pursuing interests. This may be for two reasons; one hypothesized reason is that these activities are suitable to engage in through digital means, and maybe more convenient, accessible, engaging or fun in the digital space. Another reason may be that pandemic anxiety around exposure may remain, regardless of governmental restrictions.

### Strengths and limitations

This study has several strengths including its large sample size, the longitudinal tracking of participants, mixed-method analysis, the inclusion of measures on a variety of activities during multiple lockdown phases of the COVID-19 pandemic.

However, there are several limitations which the authors acknowledge. Firstly, the study is not based on a nationally representative sample, and this is attributed to the following points–firstly, the sampling strategy (i.e., snowballing approach) we employed for this study typically uses a non-probabilistic approach for recruiting its respondents. Usually, if that element of randomness is removed put that risk of the data being not representative of the population its representing; Secondly, and in the same vein, although it does have a wide inclusion across all socio-demographic groups, the result is more of a reflection for older and high-educated females living in the south part of England. It is, therefore, possible that diverse experiences were not adequately captured which in turn could have been an artefact caused from the chosen sampling strategy. Although the socio-demographic factors are not analysed and focused on in this study, they need to be considered for understanding and applying the study results. Thirdly, since no data was taken before the pandemic, to understand Phase ‘0’ (Pre-pandemic), researchers applied an ‘in-person’ mode of access to all reported activities, but it is still not possible to objectively know if the way of individuals doing activities before the pandemic. This assumption disregards those who worked, exercised, met with people, or engaged in other activities online before social distancing demanded it. Fourthly, the phrasing of the question was posed in a relative, subjective manner comparing the change to the ‘normal’ way of doing activities before the pandemic. However, the questions were rephrased in phase 3 to directly collect the way of people doing activities other than in a comparative way. In addition, the measure did not capture what or why participants were changing their frequency and mode of doing these activities, and thus it was not possible to assess if there are other personal or social reasons contributing to the changes. Fifthly, and the last, one limitation lies in the fact that the participants tend to mostly be the elder and higher educated people, which reduces the generalisability of the findings. Notwithstanding the relatively unbalanced sample, this work offers valuable insights into the understanding of how people adapt to the COVID-19 pandemic and public health policy change and the impact of these on people’s time use and lifestyle change. Further studies could assess the long-term effects of the lifestyle change and investigate how demographic factors influenced the results.

## Conclusion

Through this population-based study which integrates a cross-sectional and a longitudinal framework, we were able to capture changes in how people spent their time during the first 15 months of the COVID-19 pandemic. Frequency and mode of access were measured across sixteen different actives and seven phases of the pandemic. For those who lived through this time, it may be unsurprising that it was found that there was a large shift in activities from online to in-person. It was also understood that the earliest stages of lockdown represented a large decrease in activities across most measured activities and pastimes, a telling exception being ‘keeping up with the news’. As we can see from the data, during the latter relaxation and implementation of subsequent lockdowns, people began to increase the frequencies of many activities, and access them online, or in a hybrid way. This may represent a certain level of capacity that we are gaining to make efforts to perform our ‘regular’ activities, whether that be a masked outdoor meeting with friends, or taking an online exercise class. While some of these changes in the middle-stages of the pandemic could be attributed to the requirements that policy placed on the public to restrict movement, socialization, and travel, we also see a sustained change after all restrictions were lifted. Many people post-Freedom Day have reported that they continue to access some activities online. This may represent a ‘new normal’ in terms of people’s willingness and literacy to access resources online. This increased literacy may be a boon for increased resilience and preparedness for the next pandemic, and equal access to online recourses will be important to ensure as future lockdowns are necessary. It will be important for future research to investigate the consequences of decreased exposure to socialization and culture has, particularly on vulnerable groups like the elderly and children.

## Supporting information

S1 File Questionnaire. (DOCX)

S2 File Alluvial plots by activity indicating individuals’ adaptations throughout the study phases among the repeated measures (N = 203). (PDF)

S1 Table Demographic details of the participants. (XLSX)

S2 Table Change of proportions between phases and statistical test results (corresponding to Figs 2–4). (XLSX)

S3 Table Statistical test result assessing change of frequency and mode for each activity among the repeated measures (corresponding to Figs 5 and 6). (XLSX)
