# Supplementary material for: To Zoom or not to Zoom: A longitudinal study of UK population’s activities during the COVID-19 pandemic
Source: PLoS One. 2022 Jul 13;17(7):e0270207. doi: 10.1371/journal.pone.0270207 (PMC9278744; doi:10.1371/journal.pone.0270207)
Supplement: S2 File — (PDF) [file pone.0270207.s002.pdf]

Supporting information 5. Alluvial plots by activity indicating individuals' adaptations throughout the study phases among the repeated measures (N=20

a) Spending time with family

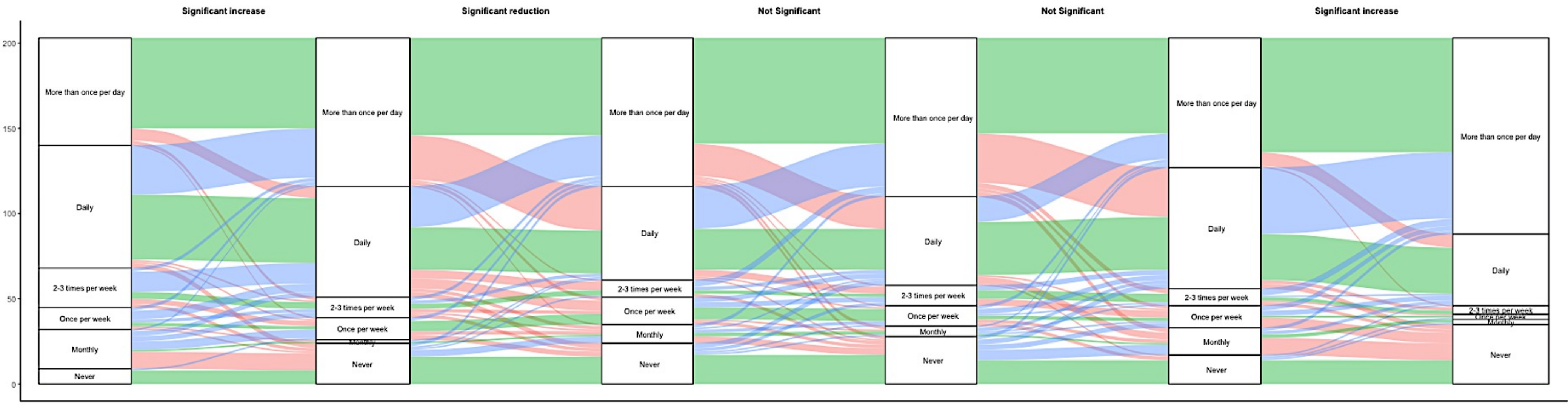

b) Spending time with others

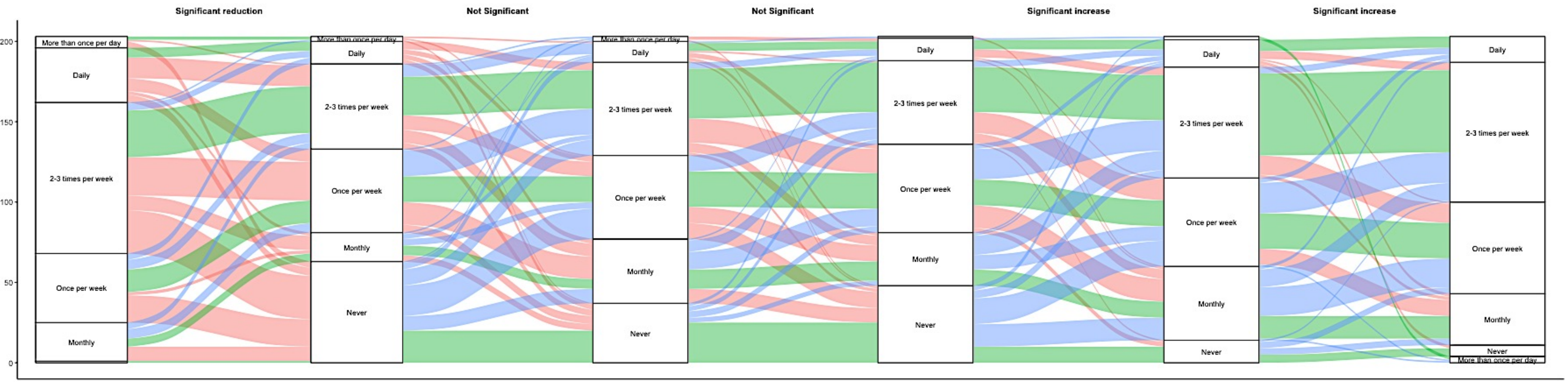

c) Relaxation

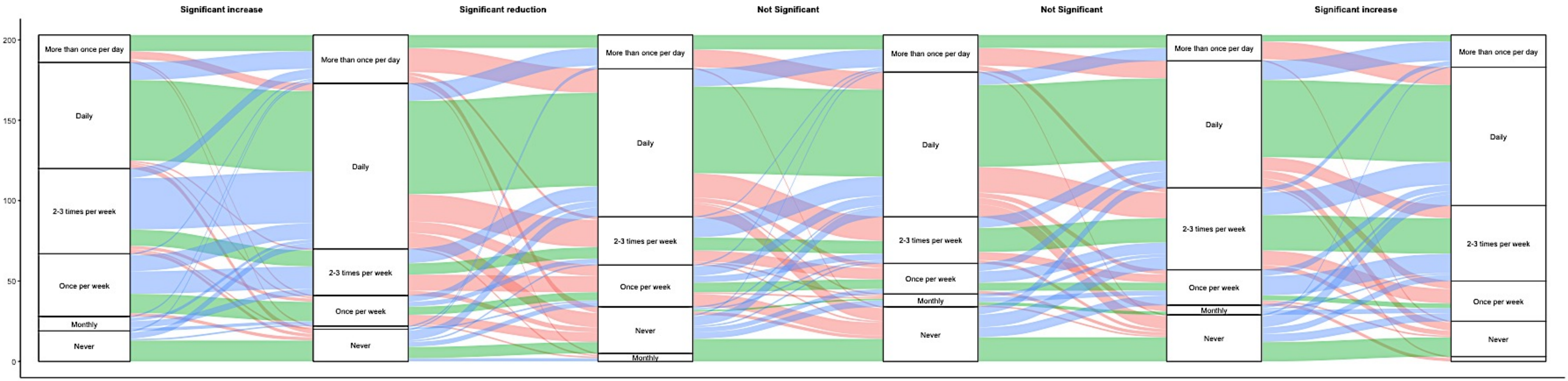

d) Getting active

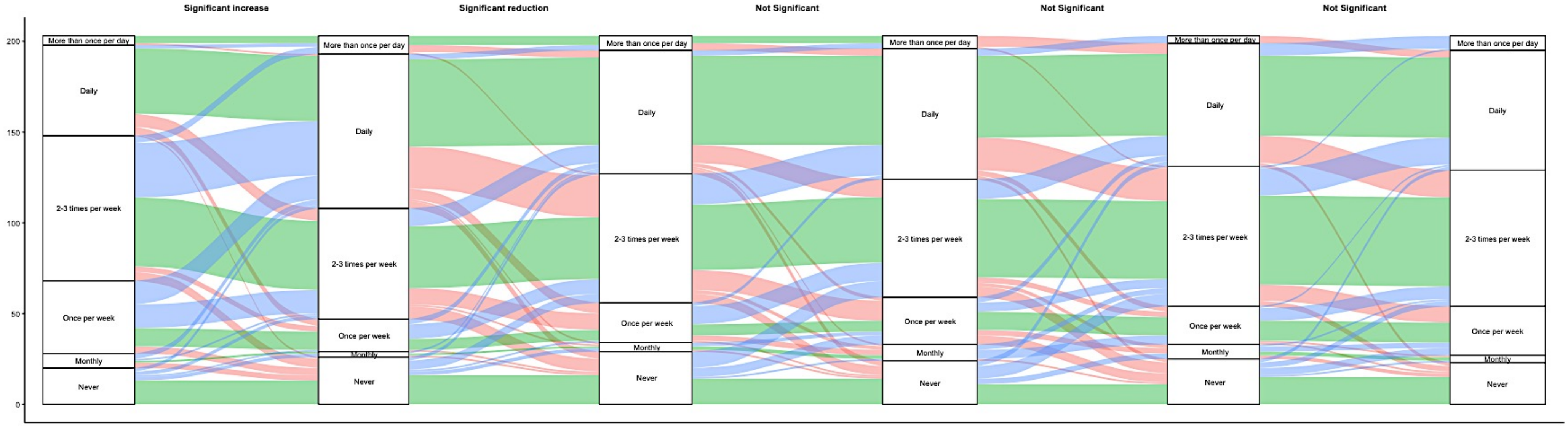

e) Interests

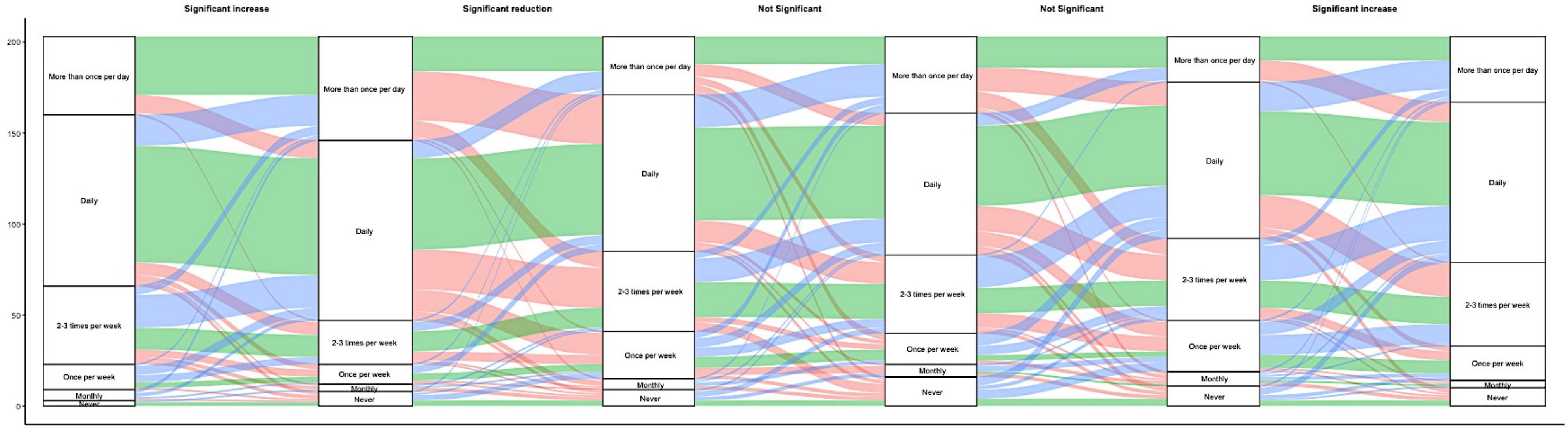

f) Journaling

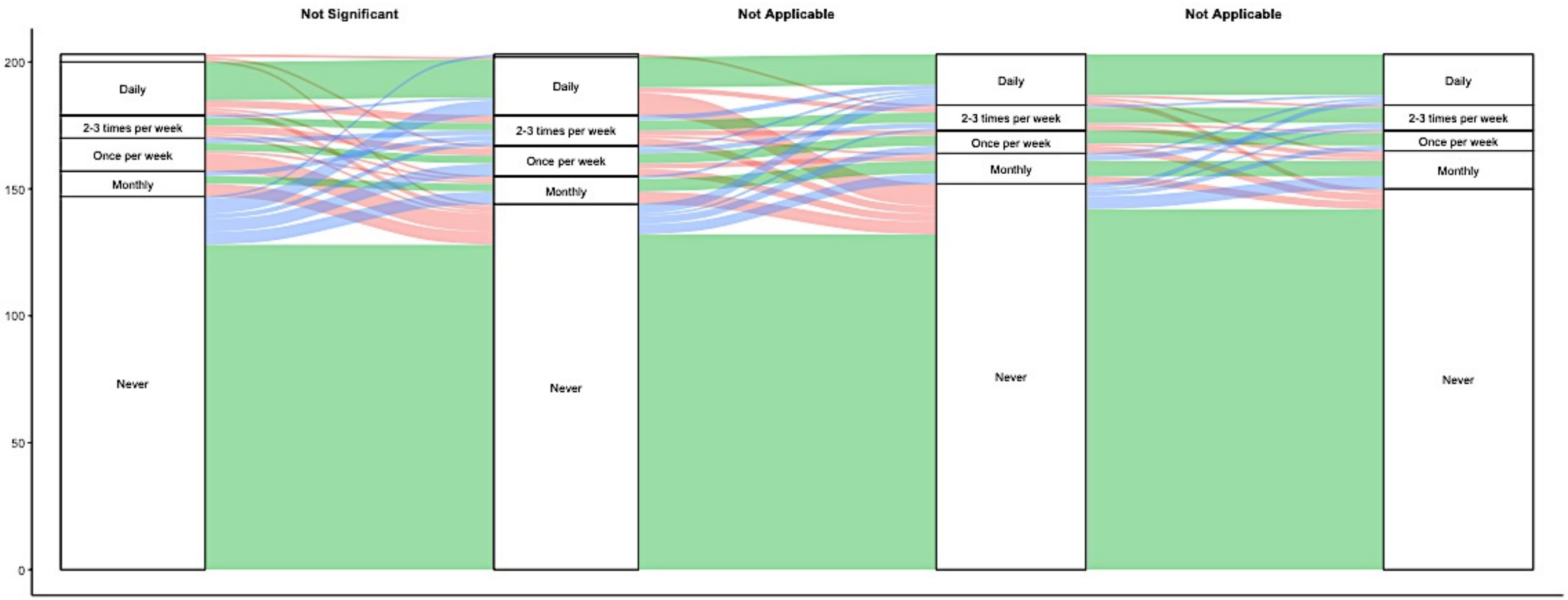

g) Social media

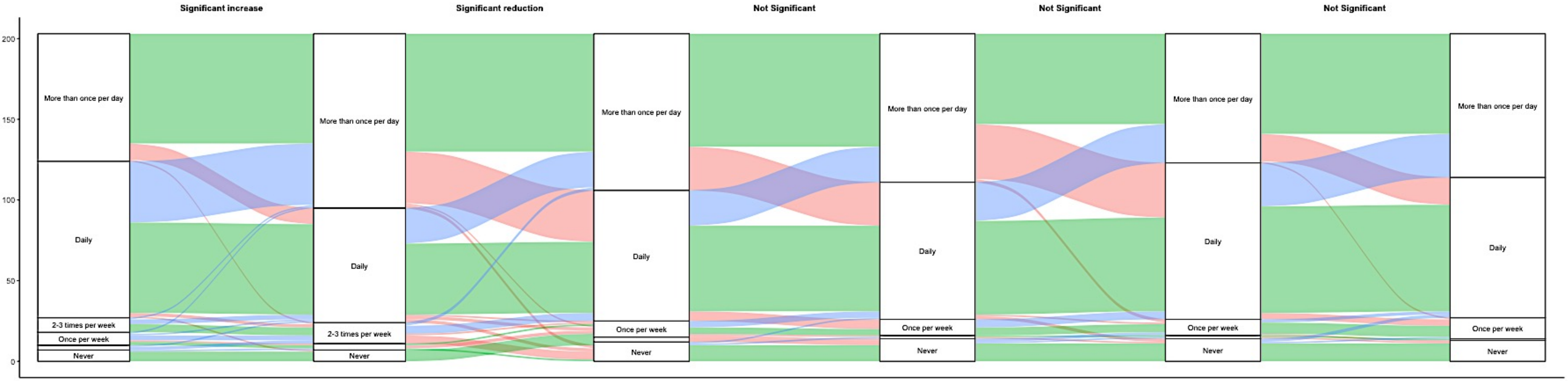

h) Home activities

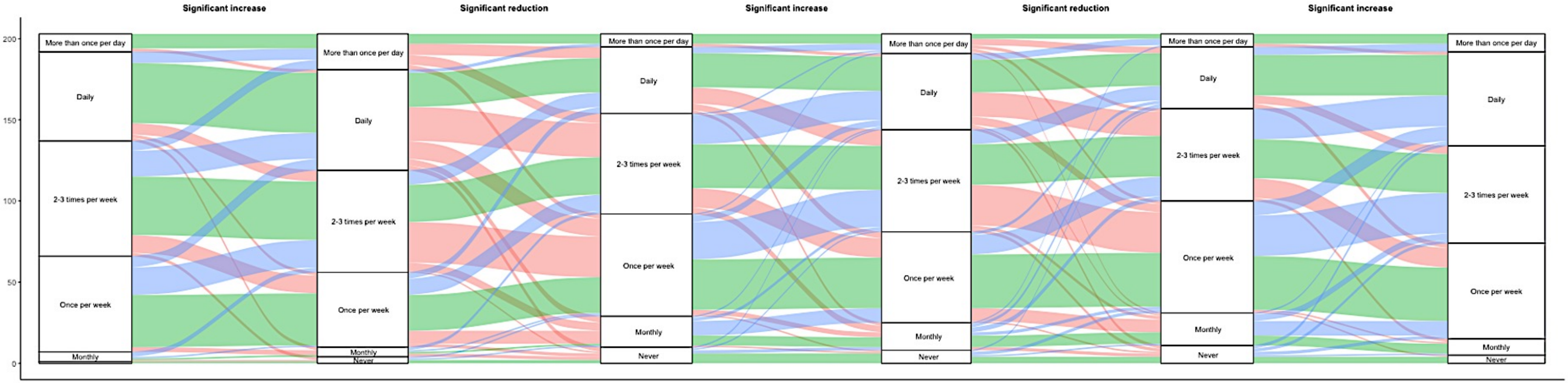

i) Shopping

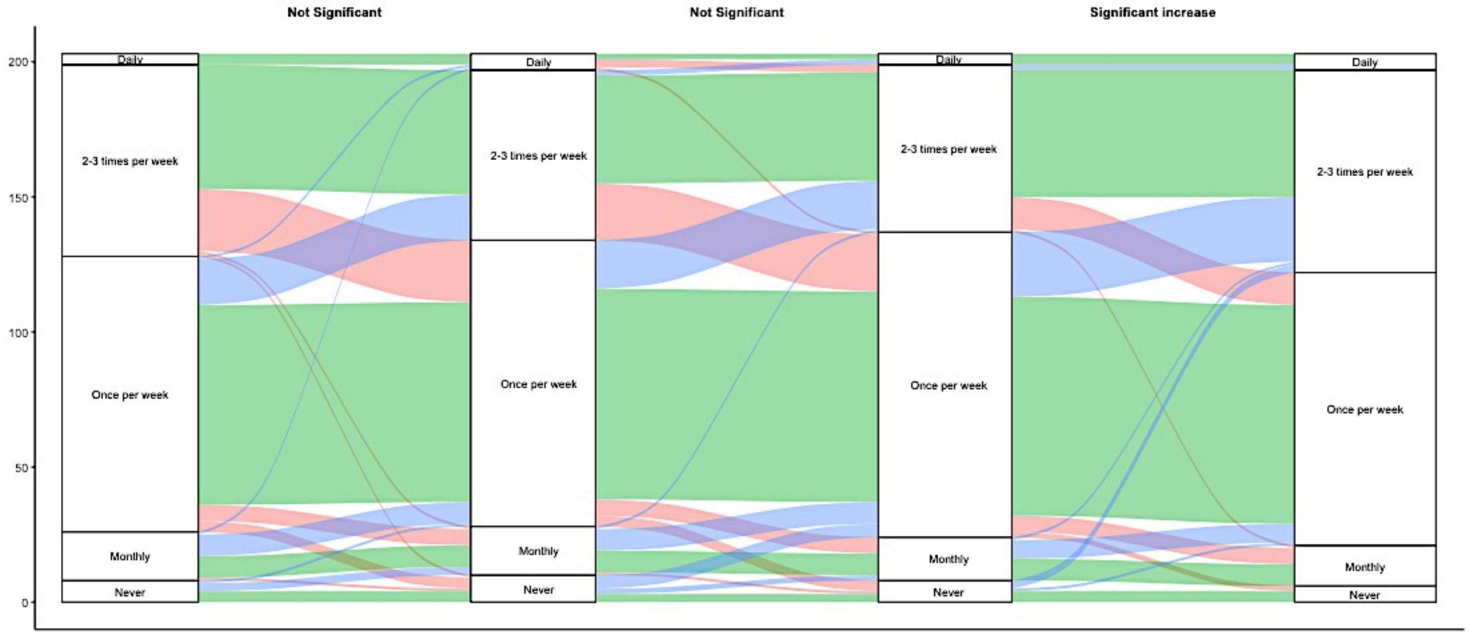

j) Work and study

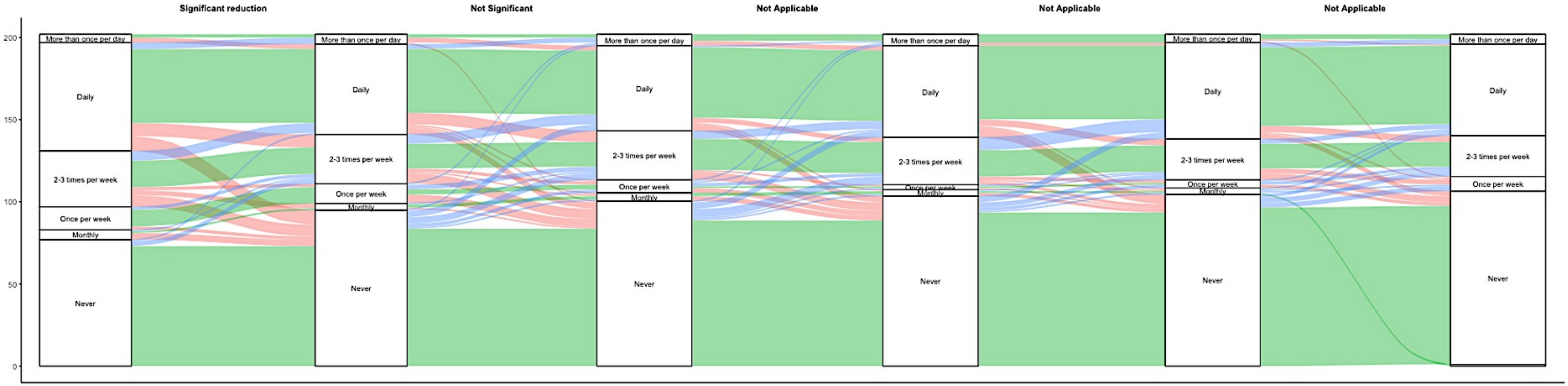

k) Travelling

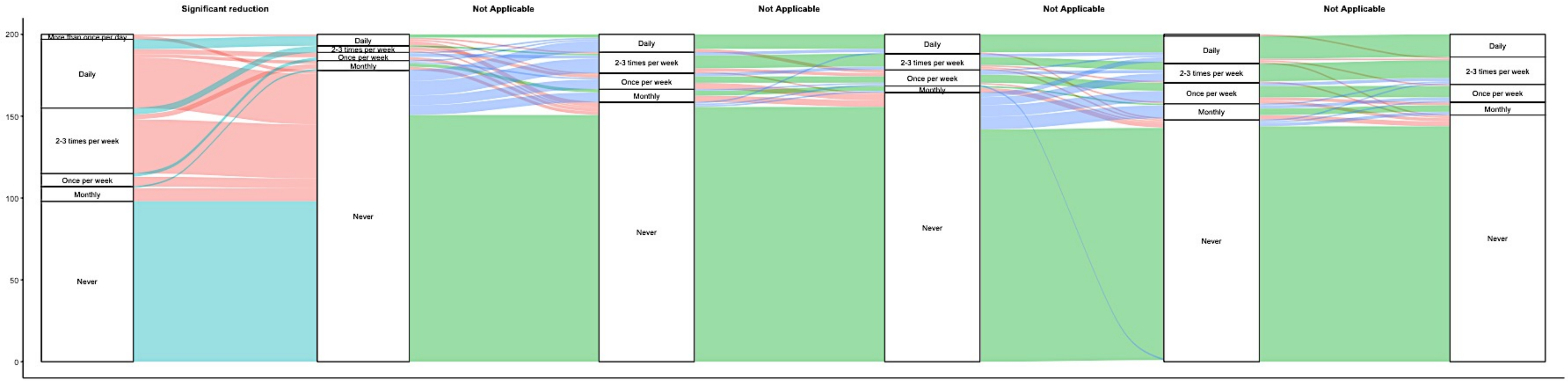

l) Helping others

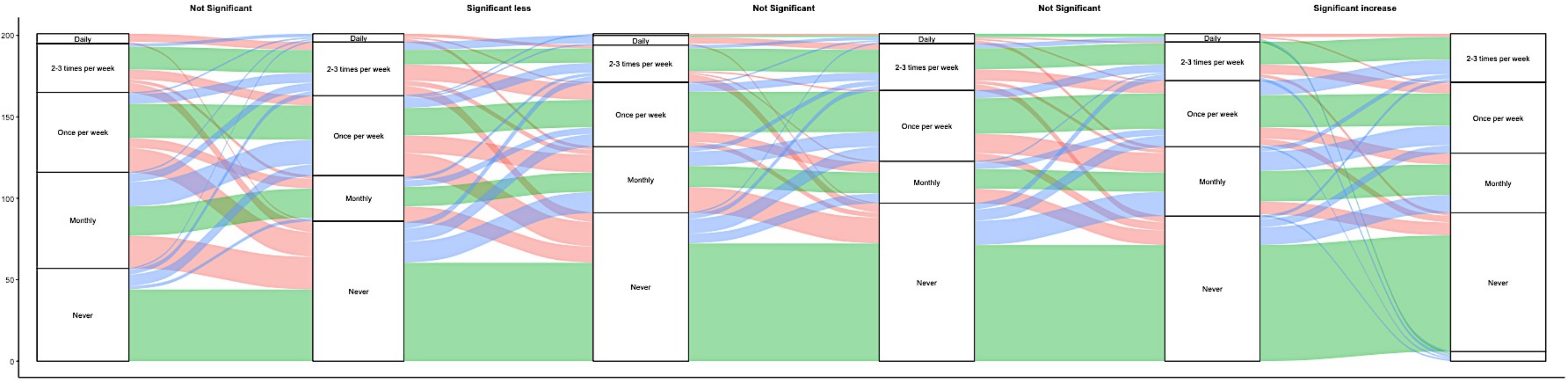

m) Pet

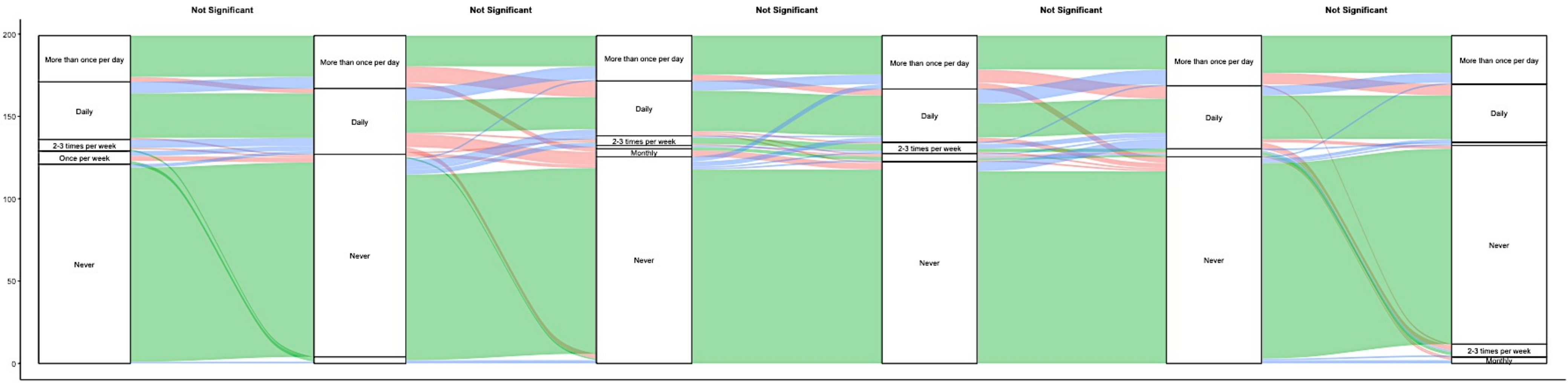
Not Significant
Not Significant
Not Significant
Not Significant
Not Significant
200
150
100
50
0
More than once per day
Daily
2-3 times per week
Once per week
Never
More than once per day
Daily
Never
More than once per day
Daily
2-3 times per week
Monthly
Never
More than once per day
Daily
2-3 times per week
Never
More than once per day
Daily
Never
More than once per day
Daily
Never
2-3 times per week
Monthly
